# Development of an In Vitro 3D Model for Investigating Ligamentum Flavum Hypertrophy

**DOI:** 10.1186/s12575-020-00132-6

**Published:** 2020-09-01

**Authors:** Cheng-Li Lin, Yi-Ting Kuo, Che-Hao Tsao, Yan-Jye Shyong, Shu-Hsien Shih, Ting-Yuan Tu

**Affiliations:** 1 Department of Orthopaedic Surgery, National Cheng Kung University Hospital, College of Medicine, National Cheng Kung University, Tainan, 70101 Taiwan; 2 Skeleton Materials and Bio-compatibility Core Lab, Research Center of Clinical Medicine, National Cheng Kung University Hospital, College of Medicine, National Cheng Kung University, Tainan, 70101 Taiwan; 3 Medical Device Innovation Center (MDIC), National Cheng Kung University, Tainan, 70101 Taiwan; 4 Department of Biomedical Engineering, National Cheng Kung University, Tainan, 70101 Taiwan; 5 Institute of Clinical Pharmacy and Pharmaceutical Sciences, National Cheng Kung University, Tainan, 70101 Taiwan; 6 International Center for Wound Repair and Regeneration, National Cheng Kung University, Tainan, 70101 Taiwan

**Keywords:** Lumbar spinal stenosis, Ligamentum flavum, Ligamentum flavum hypertrophy, 3D cell culture, Spheroid

## Abstract

**Background:**

Ligamentum flavum hypertrophy (LFH) is among the most crucial factors in degenerative lumbar spinal stenosis, which can cause back pain, lower extremity pain, cauda equina syndrome and neurogenic claudication. The exact pathogenesis of LFH remains elusive despite extensive research. Most in vitro studies investigating LFH have been carried out using conventional two-dimensional (2D) cell cultures, which do not resemble in vivo conditions, as they lack crucial pathophysiological factors found in three-dimensional (3D) LFH tissue, such as enhanced cell proliferation and cell cluster formation. In this study, we generated ligamentum flavum (LF) clusters using spheroid cultures derived from primary LFH tissue.

**Results:**

The cultured LF spheroids exhibited good viability and growth on an ultra-low attachment 96-well plate (ULA 96-plate) platform according to live/dead staining. Our results showed that the 100-cell culture continued to grow in size, while the 1000-cell culture maintained its size, and the 5000-cell culture exhibited a decreasing trend in size as the culture time increased; long-term culture was validated for at least 28 days. The LF spheroids also maintained the extracellular matrix (ECM) phenotype, i.e., fibronectin, elastin, and collagen I and III. The 2D culture and 3D culture were further compared by cell cycle and Western blot analyses. Finally, we utilized hematoxylin and eosin (H&E) staining to demonstrate that the 3D spheroids resembled part of the cell arrangement in LF hypertrophic tissue.

**Conclusions:**

The developed LF spheroid model has great potential, as it provides a stable culture platform in a 3D model that can further improve our understanding of the pathogenesis of LFH and has applications in future studies.

## Introduction

Ligamentum flavum hypertrophy (LFH) is among the main components of lumber spinal stenosis (LSS). The ligamentum flavum (LF) is located between the upper and lower vertebrae lamina, extends from the cervical vertebra to the sacral vertebra, and provides protection and stability to the spinal column. Recent findings suggest that the factors leading to LFH include age-related degeneration [1, 2] inflammation [3, 4] mechanical strain [5, 6] elastic degradation and fibrosis [7, 8]. However, the current treatments for LFH are primarily limited to invasive surgical resection, underscoring the need to explore new alternatives for novel treatments or better prevention of the occurrence of LFH.

Advances in cell culture technology have assisted in vitro investigations of LFH [9, 10]. No LF cell line is commercially available, and most studies employ primary fibroblast-like LF cells derived from patients. Researchers have employed traditional two-dimensional (2D) culture platforms and animal models. In previous studies, LF cells were cultured in monolayers and subjected to different chemical or mechanical investigations, such as the induction of different growth factor [9] conditions or application of cyclic mechanical stimuli [10]. Other studies have also attempted to employ animal models to investigate LFH [11–13]. Although the results obtained from 2D cultures have greatly contributed to the study of basic cell biology in the pathogenesis of LFH, the lack of physiological conditions may limit potential findings. In animal experiments, LFH is often considered a response to chronic illness, and an extensive amount of time is required for the development of animal models. In addition, the tetrapodal posture of rodent and rabbit models significantly differs from human bipedal manifestations, underscoring the need to develop a better in vitro model [14].

Recent work has revealed enhanced cell proliferation and pronounced cell cluster formation in LFH tissue compared with non-LFH tissue [15]. These clusters may play a key role in the progression and pathogenesis of LFH. A representative in vitro culture model could provide insights that enhance our understanding of the disease process. Compared with the aforementioned traditional 2D culture systems, in vitro three-dimensional (3D) culture systems [16, 17] may better recapitulate the cell-cell and cell-matrix interactions occurring in LF tissue [18]. However, although 3D culture systems have been widely applied in many disease models, such as cancer [19–21] cardiovascular disease [22] and wound healing [23], limited information regarding in vitro 3D LF models is available. Given that clinical observations of LFH tissue have revealed that elastic fibers decrease and degenerate while collagen increases in hypertrophic tissue [24] developing a pure cell model without adding the extracellular matrix (ECM) could help us understand the changes in the ECM composition over time.

Here, for the first time, we established a spheroid-based 3D culture of primary LFH cells as a preliminary in vitro LF model for studying LFH. The growth conditions associated with different cell densities and volumes were assessed over 14 days, and long-term maintenance up to 28 days was also examined. The phenotype of the LF spheroid model was identified via immunofluorescence using different ECM markers, including fibronectin, collagen type I, collagen type III, and elastin. The ECM protein expression in LF cells was analyzed by comparing the traditional 2D and proposed 3D cultures. Histological assessments were performed for comparisons across primary tissue and the 2D and 3D cultures. In summary, the current pioneering work demonstrates a potential new direction for the development of in vitro human models for studying LFH.

## Materials and Methods

### Primary Cell Culture

The LF tissue samples were obtained from 12 patients aged on average 60 years who underwent posterior lumbar decompression surgery because of neurological symptoms due to LSS and were unresponsive to conservative measures for at least 3 months. None of these patients received epidural or selective nerve-root blocks. The control group included patients who underwent lumbar discectomy because of a herniated intervertebral disc (HIVD). Patients with malignancy, vertebral fracture or infection were excluded from the study. The detailed in vitro primary LF expansion was performed as described in a previous report [25]. First, we attempted to obtain the entire layer of the central portion of the LF and removed the epidural fat from the LF tissues. Then, the LF tissue samples obtained from the patients were washed with 1× phosphate buffer saline (PBS) and cut into pieces. The samples were placed in 10-cm tissue culture-treated dishes (Corning, USA) for expansion culture and maintained in Dulbecco’s modified Eagle’s minimal essential medium (DMEM; Gibco, USA) containing 10% fetal bovine serum (FBS; Gibco) and 1% penicillin/streptomycin (as shown in supplementary Figure S1). The cells were grown in the presence of 5% CO_2_ at 37 °C in a humidified incubator, and the culture medium was replaced two to three times per week. For subculture, primary LF cells at 80–90% confluency were detached from the culture dish by first washing with 1× PBS and then treatment with 0.25% trypsin–(ethylenedinitrilo) tetraacetic acid (trypsin–EDTA) at 37 °C for 3 min. Then, culture medium was added to inhibit the enzymatic reaction of trypsin–EDTA. Considering the finite lifespan of primary LF cells, the sub-culture was maintained at a 1:5 ratio, and only passages under 4 were used for the experiment. For subsequent immunofluorescence and histology assays, 2D conditions were generated by placing an 18 mm × 18 mm coverglass in a 10-cm petri dish seeded with a total of 2 × 10^5^ cells (≈8000 cells per coverglass), and the cells were subsequently cultured for 24 h.

### Formation of Spheroids and Viability Assay

After the primary LF cell expansion, the cells were detached from the culture dish by using the aforementioned subculture protocol. The cells were resuspended in DMEM and diluted to 100, 1000 and 5000 cells/200 μl. To form the LF spheroids, 200 μl of cell suspension was added to each well in ultra-low attachment (ULA) 96-well round-bottom plates (Corning). The medium was replaced with fresh culture medium every 2 days. Cell viability was assessed with the LIVE/DEAD® Viability/Cytotoxicity Assay Kit (L3224; Invitrogen, USA), which provides green/red fluorescent staining for live and dead cells. Working concentrations of 2 M Calcein AM and ethidium homodimer-1 were added to the cells, and the cells were incubated in 5% CO_2_ at 37 °C for 1 h. Different cell concentrations and time series changes applied to the LF cells were observed by inverted fluorescence microscopy (DMI4000B; Leica, Germany) for bright-field and fluorescent staining. Image analysis was performed using ImageJ (NIH, USA) to calculate the volume and diameter of the spheroids (Figure S1). First, the area of the spheroids under a bright-field microscope was measured using the thresholding function in the ImageJ. We assumed that the spheroids were pure spherical, and hence, the diameter derived from the measured area could be further converted to the volume.

### Flow Cytometry

The LF spheroids were first dissociated following the method reported by Grasser et al. [26] Several spheroids were collected from a ULA 96-well plate in a centrifuge tube (15 mL). After precipitation of the spheroids, the supernatants were carefully removed and washed twice with 1× PBS. Then, 1 mL of Accutase (Merck, Germany) was added, and the spheroids were resuspended 10 times using a 1000 μL pipette with a low-retention tip. The solution was transferred to a 12-well plate, incubated at 37 °C and shaken for 10 min, and the mixture was pipetted again. A visual microscopic inspection was performed to ensure that the spheroids were dissociated into single cells. If not, the incubation and pipetting steps were repeated until the spheroids dissociated into single cells. Finally, the mixture was centrifuged and washed with 1× PBS to remove the Accutase. The centrifuged pellet was fixed in ice-cold, 70% ethanol for 24 h at − 20 °C. The cells were washed twice with PBS and resuspended in 0.5 ml of propidium iodide (PI; BD Biosciences, USA) solution (20 μg/mL) containing RNase A (0.2 mg/mL) and 0.1% Triton X-100 (Sigma-Aldrich, USA) in 1× PBS for at least 15 min in the dark. Flow cytometric analyses were performed using a FACSCalibur system (BD Biosciences).

### Immunofluorescence

For 2D cell immunofluorescence staining, cells were seeded on glass coverslips. After washing with 1× PBS, the cells were fixed in 4% paraformaldehyde (Sigma-Aldrich, USA) at room temperature for 10 min and then washed three times again with 1× PBS. Permeabilization with 1× PBS containing 0.1% Triton X-100 was performed for 20 min, followed by blocking in blocking solution (SuperBlock Blocking Buffer; Thermo Fisher Scientific, USA) for 30 min at room temperature. For 3D spheroid immunofluorescence staining, 5–10 spheroids were first collected from the 96-well ULA plate into a centrifuge tube (15 ml), and the spheroids were centrifuged in a 100 μl suspension. Then, the spheroids were placed on Polysine Adhesion Slides (Thermo Fisher Scientific) for 1 h to allow the spheroids to attach to the glass substrate. Then, the spheroids were washed twice to remove the medium and fixed using 100% ice-cold methanol at − 20 °C for 30 min. After washing three times with 1× PBS, the spheroids were blocked with an equal volume of 0.5% Triton X-100 (Sigma-Aldrich, USA) in blocking buffer at room temperature for 30 min. The spheroids were incubated with primary antibody at 4 °C overnight, washed, and then incubated with the corresponding Alexa Fluor-conjugated secondary antibodies, phalloidin-TRITC (1:2000; GeneTex, USA) and DAPI for 1 h at room temperature. The slides were mounted in glycerol-gelatin (Sigma-Aldrich) after washing. The immunofluorescence staining images were acquired by scanning confocal microscopy (TE-200; Nikon, Japan) with 408, 488, and 543 nm lasers.

### Western Blot Analysis

The cells were lysed in lysis buffer containing 1% Triton X-100, 50 mM Tris (pH 7.5), 10 mM EDTA, 0.02% NaN_3_ and a protease inhibitor mixture (Roche Diagnostics, Germany). The total protein concentration was measured by the Bradford method, and bovine serum albumin was used as a standard. Equal amounts of protein were separated by sulfate-polyacrylamide gel electrophoresis (SDS-PAGE) on 10% or 7.5% gels. The resolved proteins were transferred onto polyvinylidene fluoride (PVDF) membranes (Millipore, USA), which were then blocked with 5% nonfat dried milk in 1× PBS with 0.1% Tween® 20 detergent (PBST) for 1 h at room temperature before incubation overnight at 4 °C with primary antibodies. Then, the PVDF membrane was washed with 1× PBST before incubation for 1 h at room temperature with the secondary antibody. The gels were then scanned using a laser scanner. The proteins were quantified using densitometry with the ImageJ.

### Histology

Two consecutive sections (4-μm thick) were cut from the tissue samples (*n* = 10) by using a paraffin microtome. Several spheroids were collected from the ULA 96-well plates and washed with 1× PBS. After washing, the spheroids were processed to form 10-μm frozen sections. The spheroids were incubated in 30% sucrose in 1× PBS for 24 h at 4 °C and then embedded in optimum cutting temperature (OCT) compound before frozen sectioning. For 2D culture, LF cells (1 × 10^5^) were grown on a coverglass. Finally, the sections were stained by hematoxylin and eosin (H&E).

### Statistical Analysis

The data are presented as the mean ± standard deviation. Statistical differences were evaluated with a one-way ANOVA. A *p* value < 0.05 was considered statistically significant. The statistical analysis was conducted with Prism 8 (GraphPad Software Inc., USA).

## Results

### Aggregation volume change and viability of LF spheroids over time

**Fig. 1:**
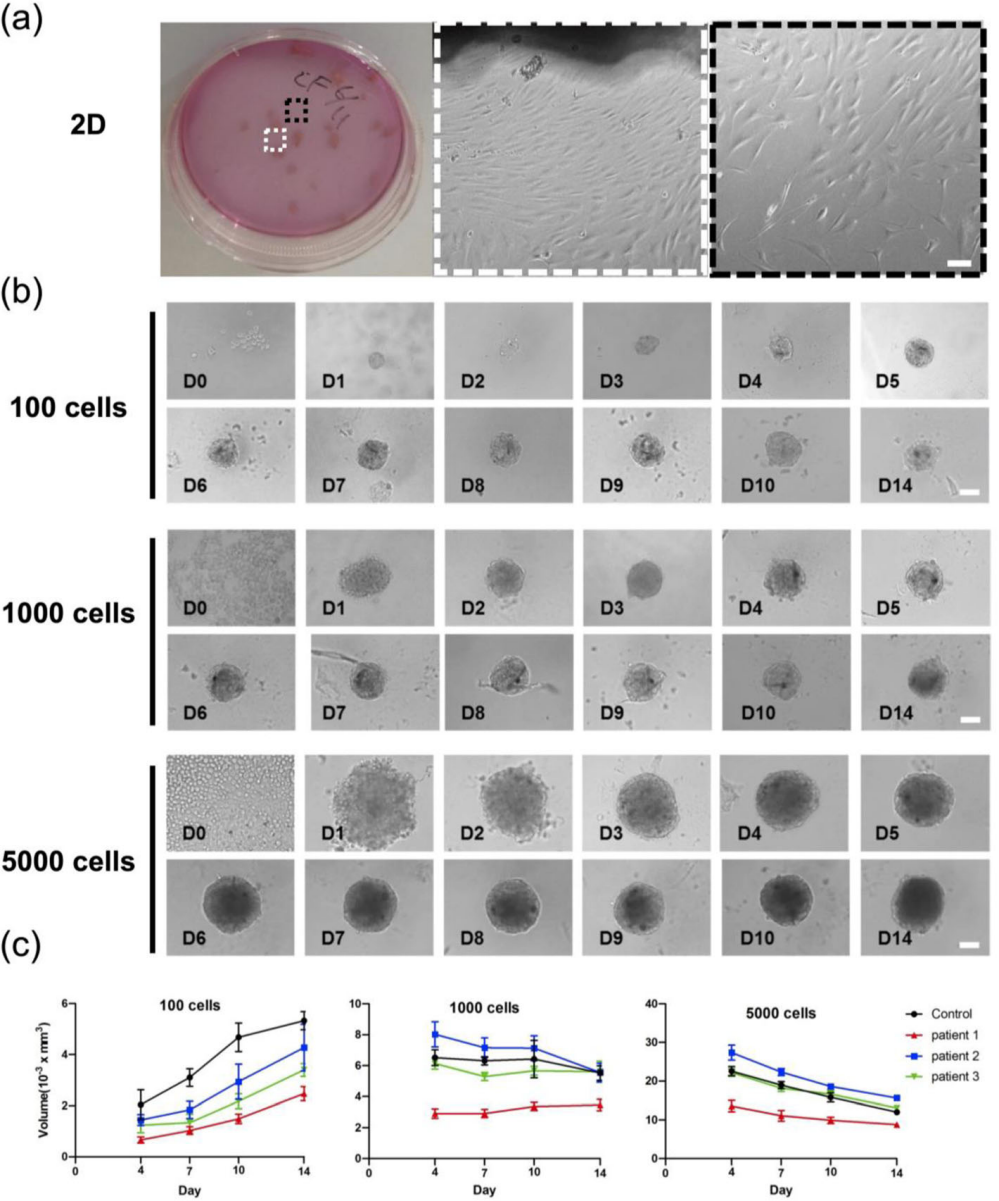
In vitro 2D LF cell expansion, aggregation processes and volume change in LF spheroids over time. **a** Minced LF tissues placed in a petri dish maintained over 3 weeks showing the cell morphology adjacent to the tissue (white dotted line) or away from the tissue (black dotted line). The scale bar is 100 μm. **b** Different cell numbers (100, 1000 and 5000 cells) were seeded in a well. Continuous observation for 14 days. The scale bar is 100 μm. **b** Quantification of LF spheroid volume on days 4, 7, 10 and 14. Four different patient samples and different cell numbers were quantified (*n* = 3)

To evaluate the growth trend during the aggregation of LF cells, both 2D in vitro expansion and ULA 96-well plates for 3D cultures at different cell seeding numbers were examined (Fig. 1). Figure 1a shows the cell morphology of minced LF tissues placed in a petri dish maintained over 3 weeks adjacent to the tissue (white dotted line) or away from the tissue (black dotted line). The LF spheroid formation process was observed using different cell numbers (100, 1000 and 5000) continuously from day 0 to day 14 (Fig. 1b). Based on the bright-field microscopy images, we found that the cells began to aggregate after 6 h (D0) of cell seeding. One hundred cells quickly formed a complete spheroid on day 1, and 1000 cells also smoothly aggregated on day 2. While the aggregation of 5000 cells was slower than that of 100 cells and 1000 cells, it still formed compact spheroids on day 4. During the 14-day period, the spheroids grown under the three different cell number conditions all became round in shape. The volume of the 1000-cell and 5000-cell spheroids was quantified on days 4, 7 and 14 (Fig. 1c). As a control group, we used cells obtained from patients with herniated intervertebral disc instead of LFH. According to the quantitative results, the size of the 100 cells gradually increased over time (≈2–4×10^− 3^ mm^3^ at day 14). Interestingly, while the size of the 1000 cells did not significantly change during the culture time (≈5×10^− 3^ mm^3^ at day 14), the size of the 5000-cell spheroids consistently decreased as the culture time increased (≈15×10^− 3^ mm^3^ at day 14). The growth trends of the LFH and control (non-LFH patient sample) groups were similar, with no distinctive differences observed.

**Fig. 2:**
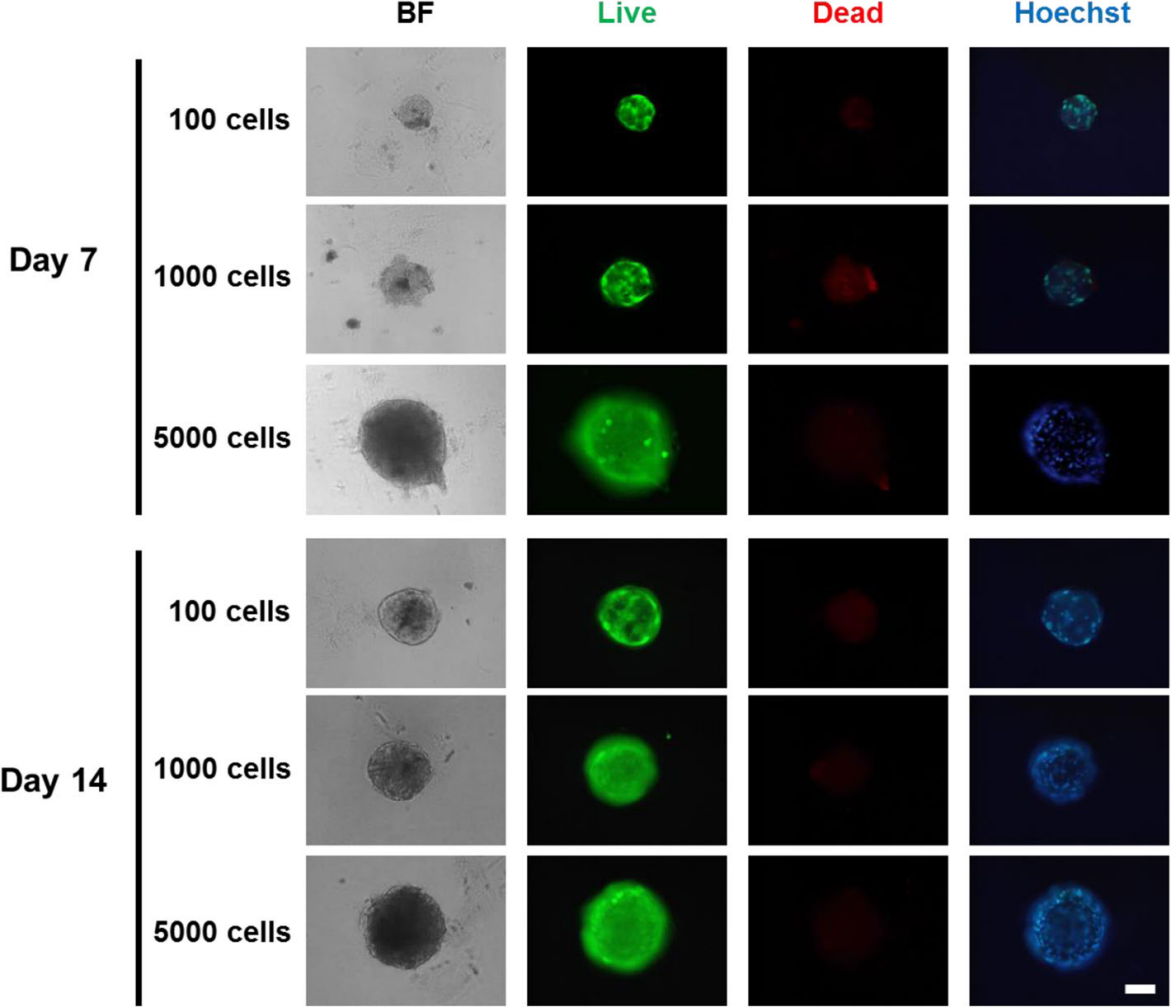
Live and dead cell staining of LF spheroids in spheroids with different cell concentrations. Spheroids were cultured for 14 days. Live and dead staining on days 7 and 14. BF represents a bright-field image. Live cells exhibit intense green fluorescence. Dead cells exhibit red fluorescence. Hoechst was used to stain the nuclei

To confirm that the ULA 96-well platform is a stable method for generating LF spheroids, we evaluated the cell viability within the spheroids (Fig. 2). The cells were cultured for 14 consecutive days and assessed on day 7 and day 14. The results showed that regardless of the cell number in the spheroids, most cells exhibited green fluorescence. Few scattered dead cells were detected with the ethidium homodimer (red) staining and could be observed in the center of the spheroids. Therefore, most LF cells in the spheroids presented good viability through day 14.

### Investigation of LF Spheroid Long-Term Culture for 28 Days

**Fig. 3:**
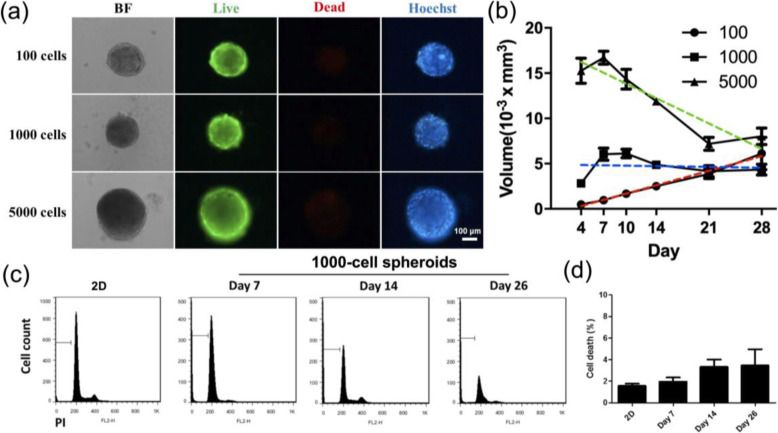
Investigation of LF spheroid long-term culture for 28 days. Morphology of LF spheroids with different cell numbers cultured for 28 days. **a** Bright-field image, live/dead cell staining, and Hoechst staining. **b** The growth trends of 100, 1000 and 5000 cells over 28 days; the linear regression of each curve is indicated as color dotted lines. **c** Cell death analysis in 2D and 1000-cell spheroids performed by flow cytometry. (4) Quantification of the percentage of cell death in 2D and 1000-cell spheroids on days 7, 14 and 26 (data of day 28 are not available)

To investigate whether LF cells are suitable for long-term culture, we tested all three concentrations of 100, 1000 and 5000 cells and cultured the cells for 28 days. The bright-field images showed that the spheroids still maintained compact aggregation (Fig. 3a). The live/dead staining results showed that most cells maintained good viability at all concentrations in the spheroids. Furthermore, the quantitative results (Fig. 3b) indicated that the cell size in the 5000-cell culture exhibited a decreasing trend over 28 days. The cell size in the 1000-cell culture remained relatively constant throughout the long-term culture. As previously described, the cells in the 100-cell culture continued to grow in size. Over the long-term observation, we found that these cells reached the same size as the cells in the 1000-cell culture on day 21 (≈4.3×10^− 3^ mm^3^) and surpassed the size of the cells in the 1000-cell culture by day 28 (≈6.1×10^− 3^ mm^3^). Similarly, while the spheroids of 5000 cells were the largest in size among all cells under the three density conditions on day 4 (≈15×10^− 3^ mm^3^), the 5000-cell spheroids gradually reduced their size to slightly pass the size of the 100-cell spheroids over 28 days (≈8.0×10^− 3^ mm^3^). Considering the lack of a significant indication of cell death based on the different cell densities using the qualitative live/dead fluorescence recording, the 1000-cell spheroids were selected as a representative group to quantitatively determine the viability of the spheroids under long-term maintenance. The PI fluorescent DNA-binding dye often used for apoptosis and cell cycle analyses [27] was used. The highlighted sub G1 region showed that a minute amount of DNA fragmentation could be found in both the 2D and spheroids on different days, while the final time point of the flowcytometric analysis was performed on day 26 due to sample availability (Fig. 3c). The quantification of cell apoptosis showed that less than 5% of the cell population was apoptotic under the conditions evaluated. These results demonstrate how spheroids containing different cell numbers gradually approach the same size. In addition, we cultured the LF cells for 28 days to ensure that the cells could be maintained in long-term culture.

### Identification of the Phenotypes of LF Cells in 2D Culture and 3D Culture Using Immunofluorescence Staining

**Fig. 4:**
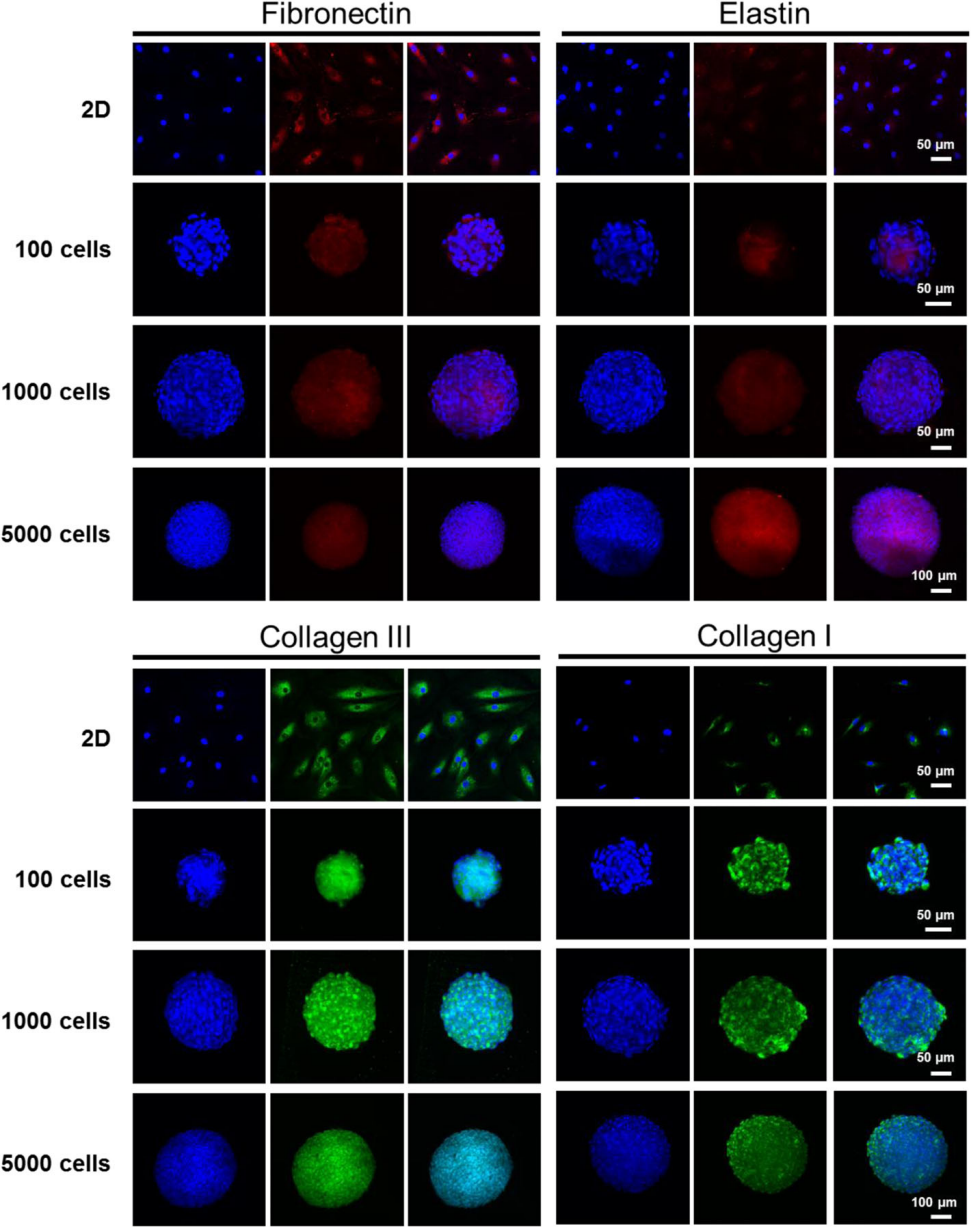
Identification of the phenotype of cultured LF cells using immunofluorescence staining. Immunofluorescence staining of fibronectin, elastin, collagen I and collagen III in 2D-cultured cells and 3D spheroids (100, 1000 and 5000 cells) was performed

LFs are fibroblast-like cells composed mainly of elastin fibers and collagen. Immunofluorescence staining was used to identify the cell phenotype of human lumbar LF cells isolated from surgical specimens obtained from patients and the cultured cells (Fig. 4). Both the 2D monolayer cells and 3D spheroids with different numbers of cells after 7 days of culture were compared. On the 2D substrate, the cells uniformly expressed fibronectin, collagen I and collagen III. Weak fluorescence of elastin was also observed in the 2D-cultured cells. The 3D spheroids expressed fibronectin and elastin, and their structure could not be clearly recognized because of the compact cell arrangement as previously mentioned. In addition, collagen I and collagen III exhibited strong fluorescence that was evenly distributed throughout the spheroid, indicating that the cultured 3D spheroid model maintained the typical phenotype after 7 days of culture. Similar results were observed in the 1000-cell spheroids cultured until day 14 (Figure S3).

### Protein Expression in LF Cells

**Fig. 5:**
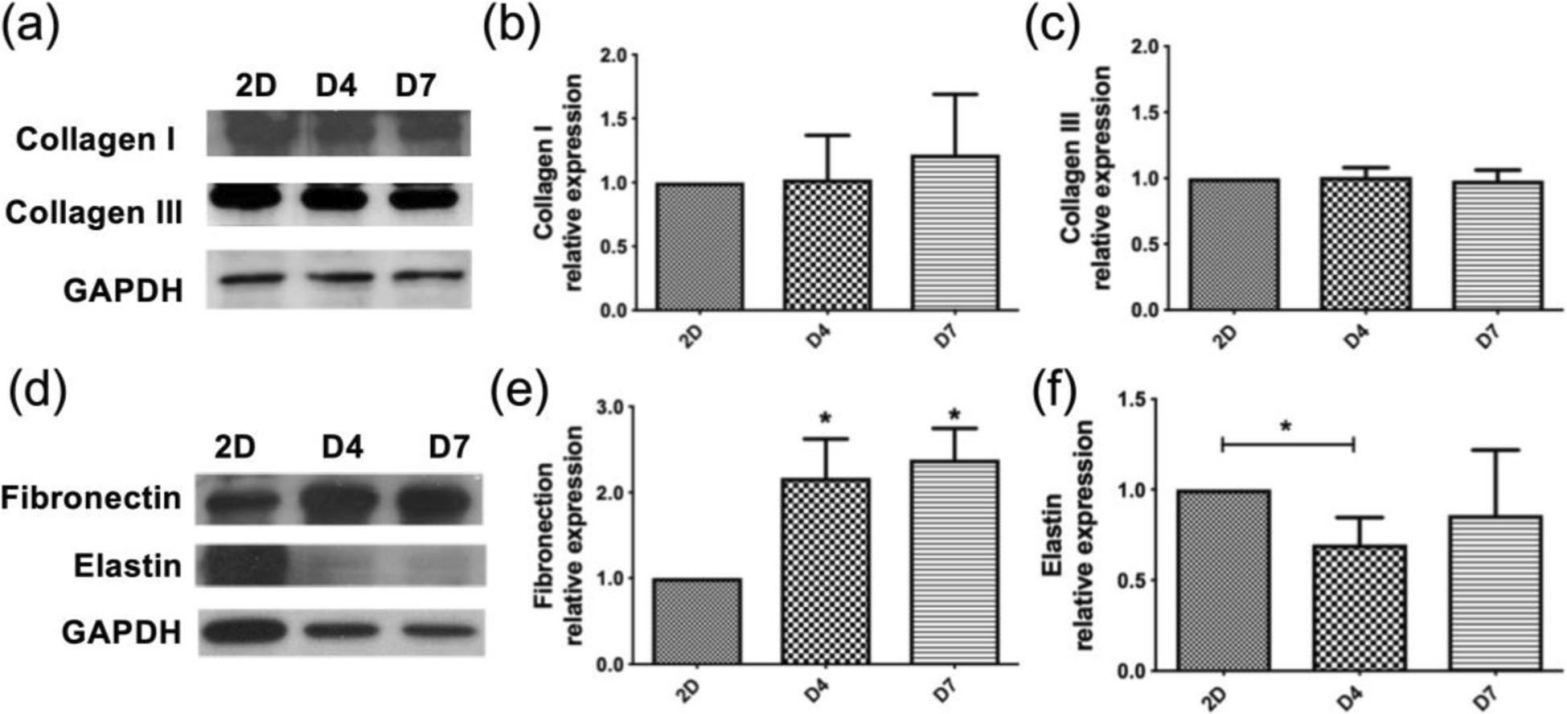
Protein expression in LF cells in 2D culture and 3D culture. One thousand spheroid cells were cultured for 7 days, and collagen I, collagen III, elastin and fibronectin were detected on days 4 and 7. **a** The expression of collagen I and III was determined by Western blotting. **b**, **c** Quantitative evaluation of (**a**). **d** The expression of fibronectin and elastin was determined by Western blotting. **e**, **f** Quantitative evaluation of (**d**). The data represent the mean ± SD. “*” denotes statistical significance with a *p* value < 0.05 among the experimental groups, whereas “**” denotes statistical significance with a p value < 0.01. Equal protein loading was verified by GAPDH

Based on the stable growth trend of the spheroids of 1000 cells in terms of both morphology and volume and the results of the cell cycle (supplement) and phenotypic expression analyses, we further investigated protein expression in spheroids of 1000 cells cultured for 7 days in comparison with the 2D cells (Fig. 5). The protein expression of fibroblast-related markers in the spheroids on days 4 and 7 was determined by Western blotting and compared with that in the 2D-cultured cells (Fig. 5a). The Western blot analysis revealed that collagen I and collagen III did not significantly change over time (Fig. 5a-c). In addition, there was no significant difference in the expression of collagen I or III between the 2D and 3D groups. We also measured the protein expression of elastin and fibronectin (Fig. 5d-f). The results showed that elastin expression in the 3D spheroids was lower than that in the 2D culture after 4 days of culture. However, elastin protein expression increased in the 3D group after 7 days. Fibronectin protein expression on day 7 in the 3D-cultured cells was significantly (*p* value < 0.05) higher than that in the 2D-cultured cells.

To further understand the components of the cell changes under different culture conditions, a Western blot analysis of ECM protein expression in the 2D-cultured cells and spheroids was performed. Normal LF tissue is composed of elastin fibers and collagen in a 2:1 ratio, while a reduction in the elastin-to-collagen ratio is apparent in LFH during aging and degeneration [8]. Both the 2D-cultured cells and 3D spheroids expressed collagen type I and III, and the collagen expression levels did not significantly differ between the two groups. Elastin expression significantly differed between the 3D spheroids and 2D-cultured cells at 4 days of culture. While elastin expression increased in the spheroids on day 7, no significant difference was observed in the 2D-cultured cells between days 4 and 7.

### Histological Comparison of Spheroids and Human LF Tissue

**Fig. 6:**
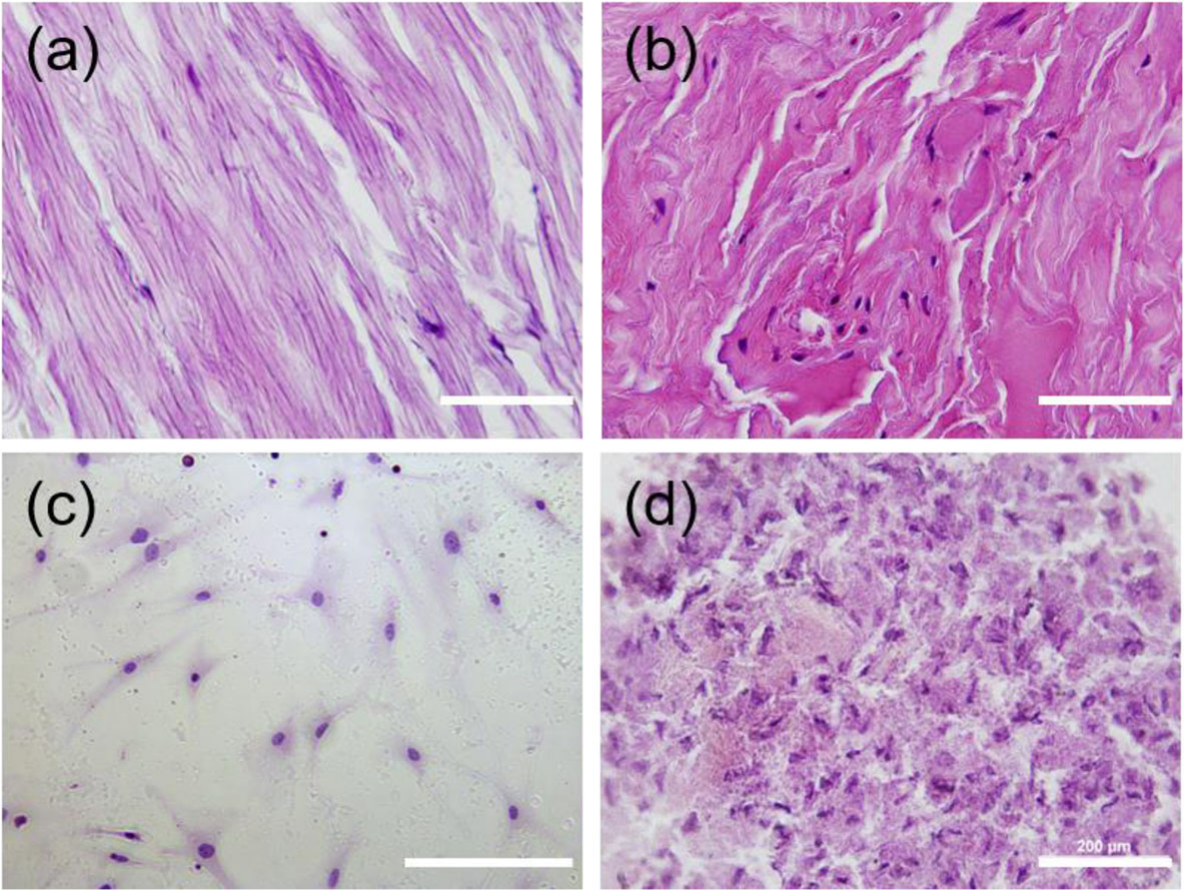
Histological comparison of spheroids and human tissue. **a** Normal LF tissue with abundant normal elastin fibers organized in parallel. **b** LFH tissue with disorganized and fragmented elastic fibers. **c** 2D LF cells were randomly distributed on the slides. **d** Frozen sections of 3D spheroids. Many nuclei could be observed and exhibited compact, nondirectional aggregation. Scale bars = 200 μm

To examine the cell structure and elastin distribution of tissue, LF cells and tissue were stained with H&E. In the normal LF tissue (Fig. 6a), a large area presented pink staining, indicating the presence of rich elastic fibers in the tissue. In addition, the elastic fibers and cells were organized in parallel. However, in the LFH tissue (Fig. 6b), several nuclei could be observed, and the elastic fibers in the LFH tissue were fragmented and disorganized compared with those in the normal tissue. The H&E staining results of the 2D-cultured cells (Fig. 6c) showed that the cells were randomly distributed on the slides, with an obvious polarized and extended morphology. Due to the difficulty of frozen tissue sectioning, the 5000-cell spheroids were selected and cultured for 4 days (Fig. 6d). In these spheroids, several nuclei in a compact structure and random aggregation were observed. In contrast to the cells in the 2D culture, the 3D-cultured cells tended to accumulate tightly in the spheroid.

## Discussion

LFH is among the most crucial factors in degenerative LSS, which not only affects the quality of daily life due to pain and discomfort but also leads to disability, especially in elderly patients. Therefore, one notable goal of this study was to provide a proof-of-concept for a spheroid cluster model for studying in vivo human LFH using an in vitro culture system with improved similarities. Primary LF cells are usually investigated in 2D cell culture on petri dishes, and thus, the characteristics of the proposed 3D spheroid culture were compared with those of conventional 2D culture.

The choice of the spheroid culture platform was based on the high reproducibility and accessibility of conventional ULA 96-well plates, which are simple to harvest for further analysis [28]. A crucial feature of ULA 96-well plates is the convenience of direct staining and observation; consequently, spheroid formation can be easily and continuously observed over time. Although the sphericity of a spheroid depends on the intrinsic properties of the cell type, LF cells seem to favor spheroid formation immediately after 1 day (Fig. 1). This rapid spheroid formation may be due to the fibroblast-like nature of LF cells; a few fibroblast-based studies have highlighted the tendency of fibroblasts to form spherical spheroids [29–31]. Another interesting aspect revealed from the time-lapse investigation was the different growth trends depending on the initial LF seeding concentration. These results may be explained from a homeostatic perspective by considering the secretion of metabolic waste, nutrient diffusion and oxygen consumption in the media. Due to the lower diffusion barrier, the 100-cell spheroids may undergo cell proliferation or ECM secretion to expand in volume over time; in contrast, the 1000-cell spheroids appeared to reach a plateau throughout the investigation period, whereas the 5000-cell spheroids primarily underwent a process of compaction, as reported elsewhere [32, 33].

Given the paucity of information regarding 3D culture of LF cells, it is imperative to ensure the viability of primary cell growth in the proposed platform (Fig. 2). Ethidium homodimer-1 binds nucleic acids in cells with damaged membranes. Only a few cells in the spheroids showed bright red fluorescence, and most cells were green, suggesting that the LF cells had good viability over the first 28-day culture period (Fig. 3). The long-term maintenance of the LF spheroids demonstrated the repeatability and reliability of the culture conditions for the growth of primary LF cells. Because the growth trends differed under distinct conditions, cell cycle [34] analysis was used to evaluate whether the different culture conditions had an effect on the cell cycle (Figure S2). Flow cytometry was performed after 4 days of 2D culture and 3D culture (Figure S2a-c). Most LF cells were in the G0/G1 phase, indicating that most cells were quiescent in both the monolayers and spheroids. However, a smaller proportion of cells were in the S and G2/M phases, indicating that the cells grew and proliferated more slowly. In the 2D culture, the cell cycle distribution did not significantly differ between the 1000-cell and 5000-cell cultures (Figure S2d). These results indicate that the cell number does not significantly affect the cell cycle of LF cells in 3D culture compared with monolayer culture. In fact, a previous study revealed that long-term culture and cloning of primary human bronchial basal cells can maintain the pluripotent differentiation capacity and cystic fibrosis transmembrane conductance regulator channel function [34]. Therefore, the long-term maintenance of in vitro primary culture provides future possibilities for functional research. We cultured LF cells for 28 days to ensure that the cells could be maintained in long-term culture.

Among the various assessment tools, morphology is the most direct method of studying the form and structure of living cells (Fig. 4). Cytoskeletal actin filaments are particularly abundant polymers under the plasma membrane that respond to environmental cues by establishing mechanical support, determining the cell shape, and facilitating cell-substrate movement [35]. Therefore, staining the cytoskeletal structure may help provide a better understanding of the peculiar shapes observed under different cell culture conditions in an intuitive manner. The results indicated that 2D planar culture on a hard plastic surface may be much less representative than spheroid culture, which provides improved cell-cell and cell-matrix interaction and 3D geometric arrangement. The expression of a matrix rich in collagen type I and III and fibronectin has been found in LFH tissue [25], and the cultured LF cells acquired an LF phenotype with a uniform expression of fibronectin, elastin and collagen types I and III under both the 2D and 3D conditions as further validated by the immunofluorescence cell staining. The initial cell seeding concentration did not appear to change the acquisition of the LF phenotype in terms of the qualitative ECM expression profile.

The results of Western blot analysis further showed the quantitative measurement of the investigated ECM proteins (Fig. 5). Fibronectin primarily differed between the 1000-cell spheroids and 2D culture, whereas collagen I and III and elastin were expressed at similar levels under both culture conditions. Because elastin degradation is an important characteristic of LFH, the minimal discrepancy in elastin expression may be attributed to the degenerative nature of the source hypertrophic tissue, underscoring the need to investigate ECM expression levels at different cell seeding concentrations and times.

Histological examination using H&E staining is a standard assessment in cytopathology used to study morphological changes, such as the arrangement of the LF structure and the severity of elastin degradation, i.e., loss, fragmentation and disorganization [36]. Compared with the nonhypertrophic LF tissue with a defined ECM orientation, the hypertrophied LF cells showed not only fragmented and disorganized fibers but also cell proliferation and cluster formation (Fig. 6). The LF cells in the LSS group displayed high elastin degradation, which is consistent with previous studies [37–39]. Notably, the morphological features of the LF cells in the 3D spheroid model were more similar to those of the LFH tissue, including random arrangement and cell-cell clustering, than those of the 2D-culture cells. In addition, based on the number of cells found in the clinical observation, i.e., approximately 10–20 cells in a slice of a cluster, each aggregation of cells in the LF tissue could reasonably be estimated to be a few hundred LF cells. This finding is also consistent with the different cell densities investigated in this report, suggesting that the 100- to 1000-cell spheroids might be related to the numbers found in vivo. However, currently, no specific conclusion regarding the optimal culture time suitable for further analysis can be drawn, but these preliminary observations highlight that this platform may provide a pan-pathophysiological in vitro model that more closely mimics LFH than conventional 2D culture.

In this study, a proof-of-concept of the generation of an in vitro 3D LF spheroid model was demonstrated using conventional ULA 96-well plates. This work is the first to attempt to develop a conducive in vitro model using a 3D culture system and report cell clustering resembling that found in primary tissue from LFH patients. These preliminary findings suggest that the growth of LF spheroids is highly dependent on the cell seeding concentration, whereas cell viability is less of a concern when culturing in ULA 96-well plates. In addition, the current platform conforms to the standard well-plate formats used by most laboratories with a high ease of control and observation. The pure cellular-based model facilitates the study of ECM expression. Thus far, due to the lack of a basic understanding of LFH, invasive surgery has remained the major avenue of treatment. Developing an in vitro model relevant to the in vivo milieu may provide insights into the field of orthopedics. Understanding the mechanism using the current platform may aid in the initiation of potential alternative treatments or the prevention of LHF.

Although the current pioneering approach demonstrated considerable robustness and potential utility, there are some limitations that should still be mentioned. First, 100-cell spheroids can be difficult to integrate with subsequent cell cycle and Western blot analyses because of the insufficiency of the cellular contents retrieved from each ULA 96-well plate. Acquiring sufficient cellular material could significantly increase the cost and involve laborious procedures. Second, the source of the primary sample greatly affects the quality of the spheroid formation. We observed that clusters could not be formed from a few primary LF cells, but the exact cause remains elusive. Third, the spheroid culture process is profoundly dependent on development over time, as the 100-cell and 5000-cell spheroids did not appear to reach a plateau or valley of growth during the 28-day investigative period. Finally, while ECM proteins were preliminary investigated, understanding the gene expression level differences between 2D and 3D cultures may provide additional information and should be considered in the scope of future studies.

## Conclusions

In this study, we established a simple 3D in vitro model of primary LF cells using ULA 96-well plates. The results indicated controllability, reproducibility and ease of observation when using the developed model and the choice of platform. Compared with the traditional 2D cultures, enhanced cell-cell and cell-matrix interactions were achieved in the 3D spheroids. The effect of the cell seeding number was correlated with the growth of the LF spheroids based on the volume. The live/dead staining results revealed that the primary LF cells showed good viability in this platform in the long-term culture. The expression of ECM proteins, including collagen I and III and elastin, was similar in the 2D- and 3D-cultured cells. Fibronectin protein expression in the 3D cultures was significantly higher than that in the 2D cultures. The histological examination suggested that the cells in the 3D culture featured disordered elastin alignment with a random distribution of nuclei, thus mimicking part of the LFH tissue. This pioneering investigation provides insights for LH research and a new avenue for alternative treatments for LFH.

## Supplementary information

**Additional file 1.**

## Data Availability

The authors confirm that the data supporting the findings of this study are available within the article and its supplementary materials.

## Abbreviations

LF: Ligamentum Flavum
LFH: Ligamentum Flavum Hypertrophy
2D: Two-dimensional
3D: Three-dimensional
ULA 96-plate: Ultra-low Attachment 96-well Plate
ECM: Extracellular Matrix
LSS: Lumbar Spinal Stenosis
HIVD: Herniated Intervertebral Disc
H&E: Hematoxylin and Eosin
