# Supplementary material for: Development of an In Vitro 3D Model for Investigating Ligamentum Flavum Hypertrophy
Source: Biol Proced Online. 2020 Sep 1;22:20. doi: 10.1186/s12575-020-00132-6 (PMC7460798; doi:10.1186/s12575-020-00132-6)
Supplement: Supplementary file 1 — Additional file 1. [file 12575_2020_132_MOESM1_ESM.docx]

Development of an *in vitro* 3D model for investigating ligamentum flavum hypertrophy

Cheng-Li Lin^1,2,3^, Yi-Ting Kuo^4^, Che-Hao Tsao^4^_,_ Yan-Jye Shyong^4,5^, Shu-Hsien Shih^1^, Ting-Yuan Tu^3,4,6*^

^1^ Department of Orthopedics, National Cheng Kung University Hospital, College of Medicine, National Cheng Kung University, Tainan 70101, Taiwan

^2^ Skeleton Materials and Bio-compatibility Core Lab, Research Center of Clinical Medicine, National Cheng Kung University Hospital, College of Medicine, National Cheng Kung University, Tainan 70101, Taiwan

^3^ Medical Device Innovation Center (MDIC), National Cheng Kung University, Tainan 70101, Taiwan

^4^ Department of Biomedical Engineering, National Cheng Kung University, Tainan 70101, Taiwan

^5^ Department of Clinical Pharmacy and Pharmaceutical Sciences, National Cheng Kung University, Tainan 70101, Taiwan

^6^ International Center for Wound Repair and Regeneration, National Cheng Kung University, Tainan 70101, Taiwan

^*^Corresponding author

KEYWORDS: lumbar spinal stenosis, ligamentum flavum, ligamentum flavum hypertrophy, 3D cell culture, spheroid

**Measurement of the spheroid volume**

First, the area of the spheroid under a bright field microscope was measured using the thresholding function in ImageJ. We assumed that the spheroids were purely spherical, and hence, the diameter derived from the measured area could be further converted to the volume.

**
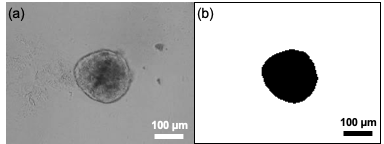
**

Figure S1. Measurement of the spheroid volume. (a) Representative bright-field image (b) Area of the spheroid after the thresholding analysis.

**Measurement of the cell cycle**

To evaluate whether different culture conditions have an effect on the cell cycle, a cell cycle analysis by flow cytometry was performed as shown in Figure S2a, b, and c. After 4 days of 2D culture and 3D culture, most LF cells were in the G0/G1 phase, indicating that most cells were quiescent in both the monolayer cells and spheroids. However, a lower proportion of cells were in the S and G2/M phases, indicating that the cells grew and proliferated more slowly. The cell cycle distribution of the 2D, 1000 cells and 5000 cells did not significantly differ, as shown in Figure S2d. These results indicate that the 3D spheroid culture does not significantly affect the cell cycle of LFs compared to monolayer cells.


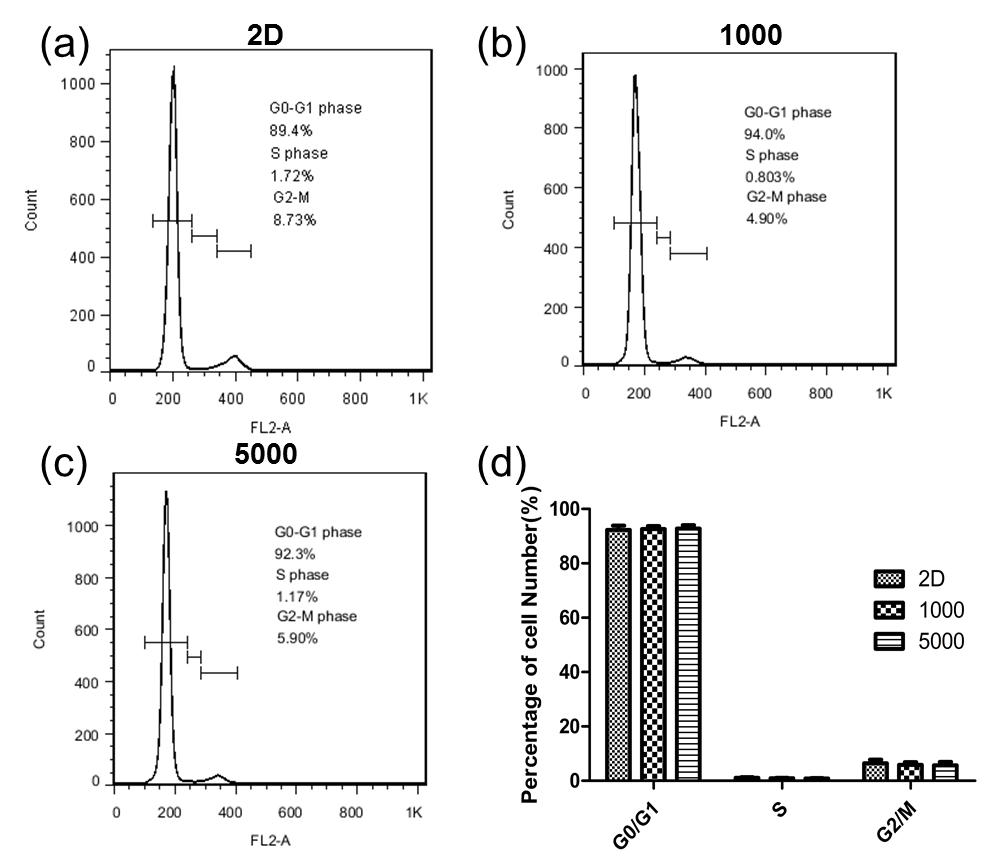


Figure S2. Cell cycle analysis by flow cytometry. (a) 2D cells (b) 3D 1000 cells (c) 3D 5000 cells (d) Cell cycle distribution of LF cells in 1000 cell, 5000 cell and 2D models.

**Identification of the phenotype of cultured LF cells using immunofluorescence staining on day 7 and day 14.**


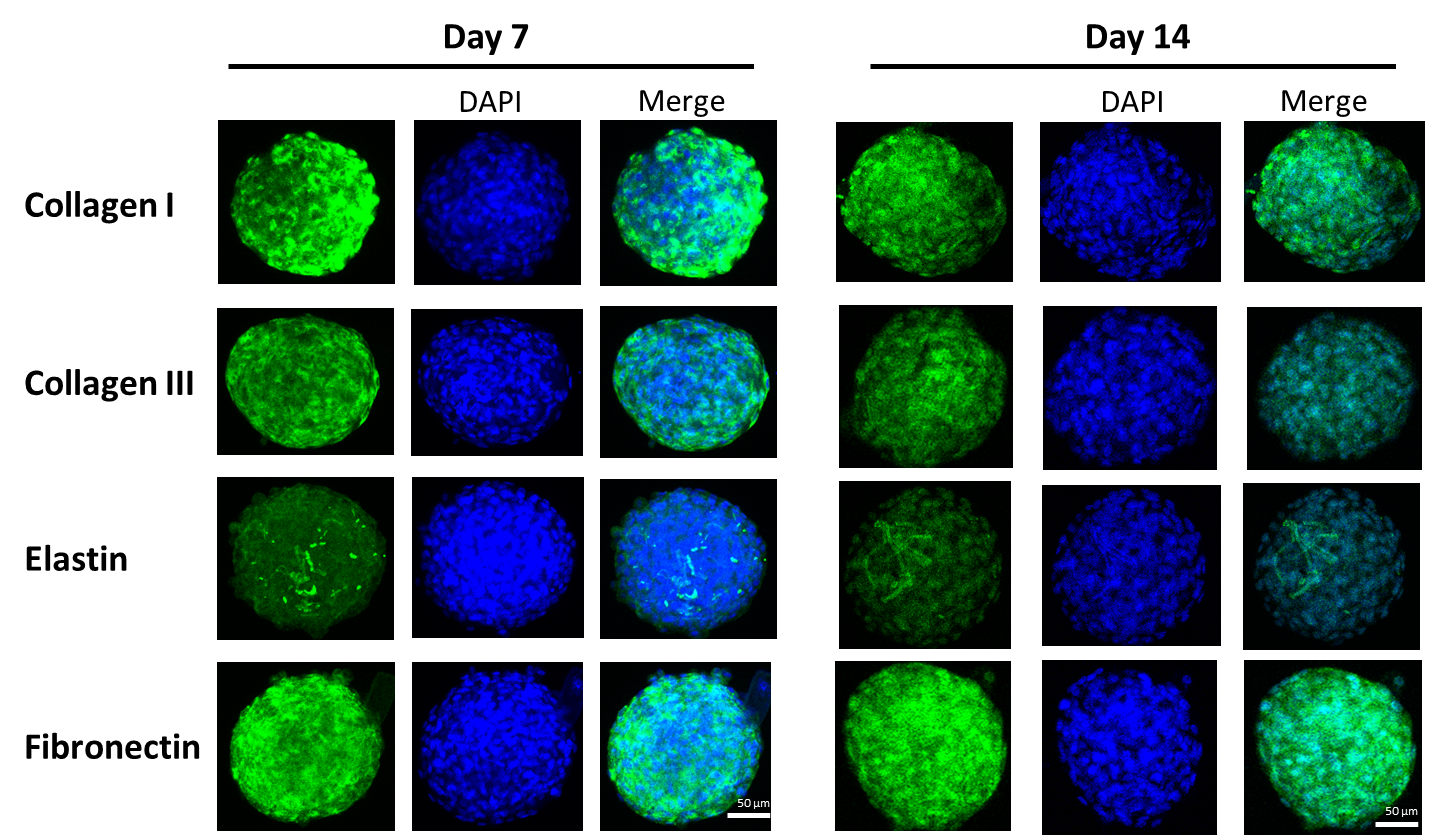


*Figure S3. Immunofluorescence staining of fibronectin, elastin, collagen I and collagen III in 1000-cell spheroids.*
